# Dietary pattern and incidence of chronic kidney disease among adults: a population-based study

**DOI:** 10.1186/s12986-018-0322-7

**Published:** 2018-12-17

**Authors:** Golaleh Asghari, Mehrnaz Momenan, Emad Yuzbashian, Parvin Mirmiran, Fereidoun Azizi

**Affiliations:** 1 Student Research Committee, Nutrition and Endocrine Research Center, Research Institute for Endocrine Sciences, Shahid Beheshti University of Medical Sciences, Tehran, Iran; 2 Nutrition and Endocrine Research Center, Research Institute for Endocrine Sciences, Shahid Beheshti University of Medical Sciences, Tehran, 1985717413 Iran; 3 Endocrine Research Center, Research Institute for Endocrine Sciences, Shahid Beheshti University of Medical Sciences, Tehran, Iran

**Keywords:** Dietary pattern, Glomerular filtration rate, Diet quality, Western dietary pattern

## Abstract

**Background & Aims:**

Although dietary patterns have been linked to chronic diseases such as cardiovascular disease, sparse data are available for a relationship between dietary patterns and incident chronic kidney disease (CKD) in West Asian populations. The aim of this study was to evaluate the association of population-based dietary pattern with the risk of incident CKD after 6.1 years of follow-up.

**Methods:**

At baseline, habitual dietary intakes of 1630 participants of the Tehran Lipid and Glucose Study (TLGS) who were free of CKD was assessed by a valid and reliable food-frequency questionnaire. The following three major dietary patterns were identified using a principal components analysis: Lacto-vegetarian dietary pattern, traditional Iranian dietary pattern, and high fat, high sugar dietary pattern. Estimated glomerular filtration rate (eGFR) was calculated, using the Modification of Diet in Renal Disease (MDRD) Study equation and CKD was defined as eGFR < 60 mL/min/1.73m^2^. Odds ratio (OR) using multivariable logistic regression was calculated for the association of incident CKD with the extracted dietary patterns.

**Results:**

After adjusting for age, sex, smoking, total energy intake, physical activity, body mass index, diabetes, and hypertension the OR for participants in the highest compared with those in the lowest tertile of the lacto-vegetarian dietary pattern was 0.57 (95% confidence interval [CI]: 0.41 to 0.80, P-trend = 0.002). In contrast, the high fat, high sugar dietary pattern was positively associated with the incidence of CKD (OR for the third tertile compared with first tertile: 1.46; 95% CI: 1.03–2.09; P-trend = 0.036). Traditional Iranian dietary pattern was not associated with incident CKD.

**Conclusion:**

The high fat, high sugar dietary pattern was associated with significantly increased (46%) odds of incident CKD, whereas a lacto-vegetarian dietary pattern may be protective against the occurrence of CKD by 43%.

**Electronic supplementary material:**

The online version of this article (10.1186/s12986-018-0322-7) contains supplementary material, which is available to authorized users.

## Introduction

Chronic kidney disease (CKD) has recently emerged as a global health burden; its worldwide prevalence has been estimated to range between 8 and 16% which could cause millions of mortalities annually [1]. Risk factors influencing CKD are divided into two main subgroups: Environment and genetics factors. Environmental factors include infections, viruses, diabetes, hypertension, obesity, oxidative stress, inflammation, and poor dietary intakes [2]. Among these, dietary intake is an important modifiable risk factor that has an association with kidney dysfunction [3–6]. In this context, most of the previous research focused only on effects of a single nutrient, food, or food group [5, 7, 8]; whereas individuals consume a variety of nutrients and foods in term of diet [9]. Hence, holistic assessment of dietary intakes by principal component analyses (PCA) provides the opportunity to consider the synergetic and interactive effect of components of diet in relation to chronic diseases, and assess many aspects of diet rather than focusing on few food groups or nutrients. Another strength of dietary-divided methods is their being population specific.

To the best of our knowledge, there is no population-based study documented on dietary patterns and CKD in the Middle East and North Africa (MENA) population. The association of extracted dietary pattern with kidney function has been examined in several studies among American [10], European [11], and Chinese populations [12]. The Western dietary pattern, characterized by red and processed meat, saturated fats and sweets, had a positive association with kidney function decline after 11 years of follow-up among participants of the Nurses’ Health Study (NHS). However, a prudent dietary pattern characterized by high intakes of fruits, vegetables, legumes, fish, poultry, and whole grains was not associated with and kidney function [10]. In addition, a study from the China Health and Nutrition Survey showed that a traditional Southern dietary pattern characterized by high intakes of rice, pork, and vegetables, and low intake of wheat increased prevalence of CKD, and a modern dietary pattern featured by high intakes of fruits, soy milk, eggs, milk, and deep fried products decreased risk of CKD [12].

Evaluating diet using a multi-dimensional and food-based approach may improve our understanding of which dietary pattern is most beneficial to prevent CKD; besides, this approach can facilitate future food-based dietary advice that is understood by the community. Therefore, we aimed to investigate the association of derived dietary patterns in our population with the incidence of CKD after 6.1-year follow-up.

## Methods

### Study population

This study was conducted within the framework of the Tehran Lipid and Glucose Study (TLGS), an ongoing population-based cohort study, aimed to identify risk factors for non-communicable diseases (NCD) in a sample of residents from District No. 13 of Tehran [13]. The baseline survey was a cross-sectional study conducted from 1999 to 2001, and surveys II (2002–2005), III (2006–2008), IV (2009–2011), and V (2012–2015) are prospective follow-up surveys.

Among the 12,523 participants examined in the third survey of the TLGS, 3462 were randomly selected for dietary assessment. Characteristics of participants who completed the food frequency questionnaire (FFQ) were similar to those of the total population in the third survey of TLGS (2006–2008) [14, 15]. Of participants who completed the FFQ, 45.4% were male compared with 44.1% in the third phase of TLGS. The percentages of the 19- to 70-year-old subjects who completed the FFQs vs the total population of the third phase were 76.7 and 82.3%, respectively. In the third phase of TLGS, 20.1% had an academic education and 11.6% were smokers compared with the 25.3 and 12.8% in subjects who completed the FFQ (Additional file 1: Table S1).

**Fig. 1:**
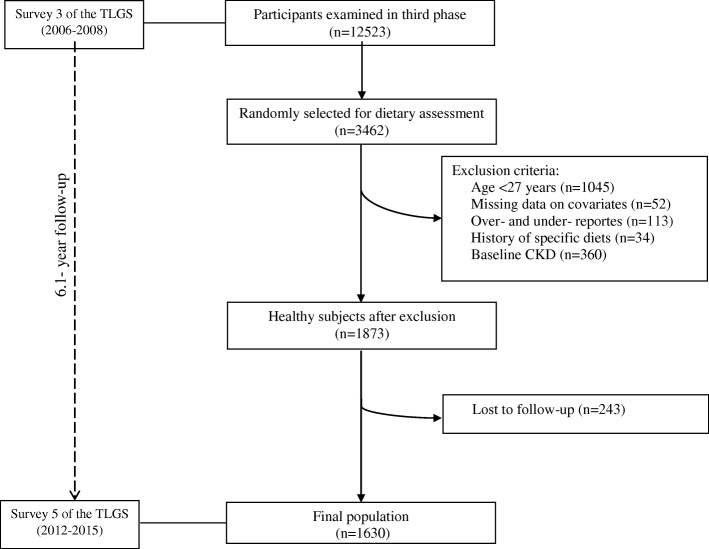
Flow chart of the Tehran Lipid and Glucose Study (TLGS) participants

In the current study, we selected 2417 participants with completed FFQ aged ≥27 years. Participants with a history of myocardial infarction or stroke and subject with possibility of major changes in diet were excluded (*n* = 34). In addition, subjects who under- or over-reported energy intakes (< 800 kcal/day or > 4200 kcal/day, respectively), (*n* = 113), those with missing data on covariates (*n* = 52) were excluded; some individuals fell into more than one exclusion category. To evaluate the incidence, we also excluded subjects who had CKD according to the Modification of Diet in Renal Disease (MDRD) equation and the national kidney foundation guidelines at baseline (*n* = 360). Finally, 1630 participants were followed until the survey fifth (2012–2015), with a median (25–75 interquartile range) 6.1 (5.6–6.5) years (response rate: 87%, Fig. 1).

The ethics committee of the institute approved the study protocol and written informed consent was obtained from all participants.

### Dietary assessment

Baseline habitual dietary intakes were assessed using a valid and reliable 168- item semi-quantitative food frequency questionnaire (FFQ) by an expert interviewer. [15, 16]. Trained dietitians during face-to-face interviews asked participants to designate their consumption frequency for each food item consumed during the previous year. As the Iranian food composition table (FCT) is incomplete, the USDA FCT was used. The reliability and validity of the FFQ evaluated against twelve 24-h dietary recalls and biomarkers in previous studies and indicated that the FFQ provides reasonably valid measure of the average long-term dietary pattern [15, 16].

### Measurement of covariates

Information on physical activity was collected at baseline using the Modifiable Activity Questionnaire (MAQ) to calculate metabolic equivalent task (MET) minutes per week. High reliability (98%) and moderate validity (47%) were found for the Persian translation of MAQ. We identified the active participants as those who had MET≥600 min/wk. [17, 18].

Weight was recorded to the nearest 100 g with subjects minimally clothed and without shoes, standing on digital scales (Seca 707; Seca Corporation, Hanover, Maryland; range 0.1–150 kg). Height was measured and recorded to the nearest 0.5 cm, when the subjects were standing without shoes, with their shoulders in a normal position. Dividing weight (kg) by square of height (m^2^), body mass index (BMI) was calculated. Arterial blood pressure (BP) was measured manually, using a mercury sphygmomanometer with a suitable cuff size for each participant after a 15-min rest in the supine position. Systolic blood pressure (SBP) was determined by the onset of the tapping Korotkoff sound, while diastolic blood pressure (DBP) was determined as the disappearance of this sound. Blood pressure was measured twice and the average was considered as the participant’s BP.

Blood samples were taken from all participants at the TLGS research laboratory after an overnight fast of 12–14 h. Fasting plasma glucose (FPG) and 2-h plasma glucose (2-hPG, equivalent to 75 g anhydrous glucose; Cerestar EP, Spain) were assayed by an enzymatic colorimetric method using glucose oxidase, with both inter- and intra-assay coefficients of variation (CVs) being less than 2%. Serum triglycerides (TGs) were assayed using an enzymatic colorimetric method with glycerol phosphate oxidase. Inter- and intra-assay coefficients of variation (CV) for TG were 0.6 and 1.6% respectively. Serum creatinine was measured at the baseline and after 6 years of follow-up, according to the standard colorimetric Jaffe_Kinetic reaction method. Both intra- and inter-assay CVs were below 3.1%; all analyses were performed using commercial kits (Pars Azmoon Inc., Tehran, Iran).

### Definitions

Hypertension was defined as SBP/DBP ≥ 140/90 mm-Hg or current therapy for a definite diagnosis of hypertension [19]. Diabetes was defined according to the criteria of the American Diabetes Association (ADA) as fasting plasma glucose≥126 mg/dl or 2-h post 75-g glucose load≥200 mg/dl or current therapy for a definite diagnosis of diabetes [20]. We used the Modification of Diet in Renal Disease (MDRD) equation formula to express eGFR in ml/min/1.73m^2^ of body surface area [21]. The abbreviated MDRD study equation is as follows:

eGFR = 186 × (Serum creatinine)^−1.154^ × (Age)^−0.203^ × (0.742 if female) × (1.210 if African − American)

Patients were classified as follows based on their eGFR levels by the national kidney foundation guidelines [22]: eGFR≥60 ml/min/1.73m^2^ as not having CKD and eGFR< 60 ml/min/1.73 m^2^ as having CKD corresponding to stages 3–5 of CKD based on the Kidney Disease Outcomes and Quality Initiative guidelines.

### Statistical analysis

We performed factor analysis (principal component factor) based on food items or groups to identify dietary patterns from the food frequency responses. This multivariate statistical technique (factor analysis) was used to reduce the complexity of diet into fewer independent factors. To simplify the interpretation, the factors were rotated by an orthogonal transformation (varimax rotation) to maintain uncorrelated factor variables called principal factor or pattern. The number of extracted factors to retain was determined by using the scree plot levels off (eigenvalues > 1.5). The factor scores of each dietary pattern for each individual was calculated by summing intakes of food items or groups weighted by their factor loading. Foods with loadings > 0.2 on a factor were used to describe dietary patterns. Dietary patterns were named according to the dominant foods in the respective patterns. Three extracted dietary pattern scores were categorized into tertiles.

Continuous variables were reported as the age- or energy-adjusted mean ± standard error and categorical variables as percentages. We calculated age-adjusted mean values for participants’ characteristics and energy-adjusted mean values for participants’ dietary intake by using analysis of covariance (ANCOVA). Tests of a trend for continuous and categorical variables across tertiles of each dietary pattern (as a median value in each tertile) were conducted using the linear regression test, respectively.

Before running multivariable linear models, the interaction terms between covariates and dietary patterns on the incidence of CKD were examined. No interaction was observed between considered covariates and dietary patterns. To examine the association of incident CKD in each tertile of an extracted dietary pattern, multivariable logistic regression models were used and odds ratio (OR) and 95% confidence intervals (CIs) were calculated. In this analysis, the first tertile of each extracted dietary pattern was considered as the reference category. To calculate the trend of ORs across increasing tertiles of extracted dietary pattern, we considered the tertile categories as continuous variables. Age (continuous), sex (male/female), smoking (current/no), total energy intake (continuous), physical activity (high/moderate/low), BMI (continuous), diabetes (yes/no), and hypertension (yes/no) were adjusted. Statistical Package for Social Sciences (SPSS) was used for data analyses and *P* values < 0.05 were considered statistically significant.

We performed several sensitivity analyses to test the robustness of the results: (1) we further adjusted for triglycerides; (2) we excluded subjects who have diabetes mellitus or hypertension at the baseline; (3) and we repeated the analysis after excluding certain population who was at stake for unstable creatinine concentrations including pregnant and lactating, extremes physical activity, BMI less than 18.5 and higher than 40 kg/m^2^.

## Results

The mean ± SD age of participants (50.5% women) was 42.8 ± 11.2 years. After 6.1 years of follow-up, we documented 220 (13.5%) cases of incident CKD with a range of eGFR as 29–59 ml/min/1.73m^2^. Mean ± SD eGFR for all participants at baseline and the end of follow up was, 73.7 ± 8.6 and 71.6 ± 11.2 ml/min/1.73 m ^2^.

Three different dietary patterns were extracted in this study based on factor analysis (Table 1). The first factor was named ‘lacto-vegetarian’ dietary patterns characterized by high intakes of fresh fruit, dried fruit and fruit juice, dark-yellow, and leafy vegetables, tomato, date, low-fat dairy, and olive oil. The second factor identified was named ‘traditional Iranian’ dietary pattern, characterized by high intakes of legumes, processed and red meat, potato, egg, refined grain, sugar, French fries, and tea. The third one was named ‘high fat, high sugar’ dietary pattern which distinguished by high intakes of mayonnaise, coffee, sweet and salty snack, soda, high-fat dairy, pizza, butter, salt, solid oil, poultry, and corn and peas. The first of the three dietary patterns accounted for 8.4, 6.7, and 5.4%, respectively, of the variance in food intake. Together, they explained 20.4% of the variability.

**Table 1:** Factor loading matrix for major dietary patterns identified by factor analysis among the population

Food Items	Factor 1	Factor 2	Factor 3
Fruit with β-carotene	0.666	–	–
Other vegetables	0.662	–	–
Fruit with flavonoid	0.631	–	–
Dark-yellow vegetable	0.550	–	–
Tomato	0.498	–	–
Other fruit	0.497	–	–
Leafy vegetable	0.435	–	–
Date	0.399	–	–
Low-fat dairy	0.374	–	–
Olive oil	0.364	–	–
Fruit juice	0.349	–	–
Dried fruit	0.256	–	–
Legumes	–	0.744	–
Processed meat	–	0.699	–
Potatoes	–	0.414	–
Egg	–	0.377	–
Red meat	–	0.343	–
Refined grains	–	0.300	–
Sugar	–	0.295	–
French fries	–	0.258	–
Tea	–	0.239	–
Mayonnaise	–	–	0.638
Coffee	–	–	0.515
Sweet and salty snack	–	–	0.490
Soda	–	–	0.451
High-fat dairy	–	–	0.335
Pizza	–	–	0.304
Butter	–	–	0.293
Salt	–	–	0.268
Solid oil	–	–	0.246
Poultry	–	–	0.220
Corn and peas	–	–	0.211
Whole grains	–	–	–
Fish	–	–	–
Non-hydrogenated oil	–	–	–

Participants in the highest tertile of the lacto vegetarian dietary pattern were younger and less likely to be male, smokers, and had lower prevalence of diabetes and hypertension, compared to the lowest tertile. The percentage of women decreased by increasing adherence to the traditional Iranian dietary pattern. Participants with higher adherence to high fat, high sugar dietary pattern tended to be older, had higher BMI, were less likely to smoke, had higher prevalence of diabetes and hypertension (Table 2).

**Table 2:** Age-adjusted baseline characteristics of participants according to the tertiles of extracted dietary patterns^a^

	Tertiles of extracted dietary patterns			
	T1	T2	T3	*P* ^b^
Lacto vegetarian dietary pattern				
Age (years)	44.6 ± 0.5	44.2 ± 0.5	41.3 ± 0.5	0.001
Men (%)	56.1	51.5	44.8	0.001
Body mass index (kg/m^2^)	28.0 ± 0.2	27.6 ± 0.2	27.2 ± 0.2	0.020
Current smoker (%)	13.3	10.2	8.2	0.041
Sedentary (%)	71.5	68.2	73.8	0.212
Diabetes (%)	8.0	6.4	3.9	0.004
Hypertension (%)	17.1	13.8	12.6	0.036
Fasting plasma glucose (mg/dl)	94.4 ± 1.0	93.0 ± 1.0	92.4 ± 1.0	0.328
Triglycerides (mg/dl)	152.2 ± 3.7	149.8 ± 3.7	155.3 ± 3.73	0.576
eGFR (ml/min/1.73m^2^)	73.3 ± 0.34	73.7 ± 0.34	74.1 ± 0.34	0.189
Traditional Iranian dietary pattern				
Age (years)	43.5 ± 0.5	43.8 ± 0.5	42.7 ± 0.5	0.290
Men (%)	52.8	53.5	46.1	0.024
Body mass index (kg/m^2^)	27.9 ± 0.2	27.6 ± 0.2	27.5 ± 0.2	0.294
Current smoker (%)	10.6	9.8	11.4	0.523
Sedentary (%)	71.5	72.4	69.7	0.717
Diabetes (%)	5.9	6.5	5.9	0.853
Hypertension (%)	13.9	15.3	14.3	0.856
Fasting plasma glucose (mg/dl)	92.3 ± 0.98	92.8 ± 0.98	94.5 ± 0.99	0.329
Triglycerides (mg/dl)	150.7 ± 3.7	150.8 ± 3.7	155.8 ± 3.7	0.543
eGFR (ml/min/1.73m^2^)	73.6 ± 0.34	73.4 ± 0.34	74.1 ± 0.34	0.361
High fat, high sugar dietary pattern				
Age (years)	39.7 ± 0.5	43.3 ± 0.5	47.1 ± 0.5	0.001
Men (%)	42.9	50.4	59.0	0.001
Body mass index (kg/m^2^)	27.4 ± 0.2	27.4 ± 0.2	28.2 ± 0.2	0.005
Current smoker (%)	15.2	10.4	6.2	0.011
Sedentary (%)	72.0	73.3	68.3	0.197
Diabetes (%)	3.2	5.3	9.8	0.001
Hypertension (%)	9.8	13.0	20.7	0.001
Fasting plasma glucose (mg/dl)	92.7 ± 1.0	92.4 ± 0.9	94.7 ± 1.0	0.217
Triglycerides (mg/dl)	151.6 ± 3.7	153.9 ± 3.7	151.6 ± 3.8	0.876
eGFR (ml/min/1.73m^2^)	73.8 ± 0.3	73.9 ± 0.3	72.4 ± 0.34	0.576

^a^Data represented as age-adjusted mean ± SE for continuous variables (except age) or percent for categorical variables

^b^Linear regression was used for continuous variables and logistic regression for categorical variables

In the energy-adjusted models, participants in the highest tertile of the lacto-vegetarian dietary pattern have less intake of total fat and sodium than those in lowest ones; they had also higher intakes of protein, carbohydrate, calcium, magnesium, potassium, and vitamin C. Participants who had higher adherence to the traditional Iranian dietary showed higher intakes of protein and carbohydrates and lower consumption of calcium, potassium, and vitamin C. The high fat, high sugar dietary pattern was characterized by higher intakes of total fat and saturated fatty acid and lower consumption of protein, carbohydrate, calcium, magnesium, potassium, and vitamin C (Table 3).

**Table 3:** Energy-adjusted baseline dietary intakes of participants according to the tertiles of extracted dietary patterns^a^

	Tertiles of extracted dietary pattern			
	T1	T2	T3	*P* ^b^
Lacto vegetarian dietary pattern				
Total energy (kcal)	2021 ± .30.1	2189 ± 30.2	2631 ± 30.2	< 0.001
Total fat (% energy)	31.5 ± 0.3	31.0 ± 0.2	30.1 ± 0.3	0.002
Saturated fatty acids (% energy)	10.6 ± 0.2	10.5 ± 0.2	9.99 ± 0.23	0.055
Protein (% energy)	13.1 ± 0.1	13.8 ± 0.1	14.3 ± 0.1	< 0.001
Carbohydrate (% energy)	57.0 ± 0.3	57.56 ± 0.3	59.26 ± 0.31	< 0.001
Calcium (mg/d)	1063 ± 17.4	1278 ± 17.1	1408 ± 17.7	< 0.001
Magnesium (mg/d)	349 ± 3.6	381 ± 3.5	421 ± 3.6	< 0.001
Sodium (mg/d)	4866 ± 134	4336 ± 132	4103 ± 136	< 0.001
Potassium (mg/d)	3005 ± 35.6	3693 ± 35.0	4734 ± 36.2	< 0.001
Vitamin C (mg/d)	83.0 ± 2.8	132 ± 2.8	226 ± 2.9	< 0.001
Traditional Iranian dietary pattern				
Total energy (kcal)	1856 ± 27.6	2203 ± 27.6	2791 ± 27.6	< 0.001
Total fat (% energy)	30.9 ± 0.3	30.8 ± 0.2	30.9 ± 0.3	0.971
Saturated fatty acids (% energy)	10.5 ± 0.2	10.3 ± 0.2	10.2 ± 0.2	0.368
Protein (% energy)	13.3 ± 0.1	13.7 ± 0.1	14.1 ± 0.1	< 0.001
Carbohydrate (% energy)	57.2 ± 0.3	57.9 ± 0.3	58.6 ± 0.3	0.006
Calcium (mg/d)	1277 ± 19	1265 ± 18	1208 ± 19	0.021
Magnesium (mg/d)	379 ± 4.0	386 ± 3.7	387.1 ± 4.1	0.206
Sodium (mg/d)	3792 ± 139	4402 ± 131	5112 ± 143	0.230
Potassium (mg/d)	3885 ± 47	3746 ± 44	3803 ± 49	< 0.001
Vitamin C (mg/d)	169 ± 3.8	142 ± 3.6	130 ± 3.9	< 0.001
High fat, high sugar dietary pattern				
Total energy (kcal)	1892 ± 28.1	2185 ± 28.1	2774 ± 28.1	< 0.001
Total fat (% energy)	27.8 ± 0.2	30.8 ± 0.2	34.1 ± 0.3	< 0.001
Saturated fatty acids (% energy)	9.11 ± 0.2	10.2 ± 0.2	11.7 ± 0.2	< 0.001
Protein (% energy)	14.5 ± 0.1	13.7 ± 0.1	13.06 ± 0.1	< 0.001
Carbohydrate (% energy)	60.8 ± 0.3	57.8 ± 0.2	55.1 ± 0.3	< 0.001
Calcium(mg/d)	1347 ± 18	1281 ± 17	1121 ± 19	< 0.001
Magnesium(mg/d)	434 ± 3.6	384 ± 3.4	334 ± 3.7	< 0.001
Sodium (mg/d)	4360 ± 139	4258 ± 132	4688 ± 143	0.143
Potassium (mg/d)	4167 ± 46	3795 ± 43	3472 ± 47	< 0.001
Vitamin C (mg/d)	158 ± 3.8	145 ± 3.7	138.2 ± 3.9	< 0.001

^a^Data represented as energy-adjusted mean ± SE for variables (except total energy)

^b^Linear regression was used for continuous variables and logistic regression for categorical variables

In Table 4, after adjusting for age, sex, smoking, total energy intake, physical activity, and BMI, the OR for subjects in the highest, compared with the lowest tertile of the lacto-vegetarian dietary pattern was 0.54 (0.39–0.76). After further adjustment for diabetes and hypertension, the OR for participants in the highest compared with the lowest tertile of the lacto-vegetarian dietary pattern was 0.57 (0.41–0.80). In contrast, after controlling for potential confounders, the high fat, high sugar dietary pattern was positively associated with incident CKD (OR = 1.46 (1.03–2.09) and a significant increasing linear trend was noted across tertiles of the high fat, high sugar dietary pattern for risk of incident CKD (P for trend = 0.036).

**Table 4:** Odds Ratio and 95% confidence intervals of incident chronic kidney disease according to tertiles of extracted dietary patterns

	Tertiles of extracted dietary patterns			
	T1	T2	T3	*P* for trend^c^
Lacto vegetarian dietary pattern				
Odds ratio for CKD^a^	Ref.	0.81 (0.60–1.09)	0.54 (0.39–0.76)	< 0.001
Odds ratio for CKD^b^	Ref.	0.85 (0.62–1.15)	0.57 (0.41–0.80)	0.002
Traditional Iranian dietary pattern				
Odds ratio for CKD^a^	Ref.	1.29 (0.95–1.75)	0.93 (0.65–1.34)	0.796
Odds ratio for CKD^b^	Ref.	1.26 (0.93–1.72)	0.91 (0.64–1.32)	0.698
High fat, high sugar dietary pattern				
Odds ratio for CKD^a^	Ref.	1.27 (0.90–1.78)	1.73 (1.22–2.44)	0.018
Odds ratio for CKD^b^	Ref.	1.21 (0.87–1.70)	1.46 (1.03–2.09)	0.036

^a^Adjusted for age, sex, smoking, total energy intake, physical activity, and body mass index

^b^Additionally adjusted for diabetes and hypertension

^c^To calculate the trend of OR across increasing tertiles of each extracted dietary pattern, we considered the tertiles categories as continuous variables (median values)

### Sensitivity analysis

Sensitivity analysis: Results were not substantially changed when we further adjusted for triglycerides. In the sensitivity analysis, that excluded subjects with hypertension and diabetes, the results showed no substantial changes (OR of the highest compared to the lowest tertile of lacto-vegetarian dietary pattern and high fat, high sugar dietary pattern: 0.67 and 1.55, respectively). Furthermore, when we excluded participants at prone to unstable creatinine concentrations, adjusted ORs for participants in the highest compared with the lowest tertiles of lacto-vegetarian dietary pattern were 0.69 (95% CI: 0.48 to 0.97), and for high fat, high sugar dietary pattern were 1.43(1.03–1.99); again there were no substantial changes in the results. On analyzing the association of incident CKD with traditional; dietary pattern score, the odds of incident CKD continued to be statistically non-significant in the multivariable model (Table 5).

**Table 5:** Sensitivity analysis: odds ratio and 95% confidence intervals of incident chronic kidney disease according to tertiles of dietary patterns

	Tertiles of extracted dietary patterns			
	T1	T2	T3	*P* for trend^d^
Sensitivity analysis^a^				
Lacto vegetarian dietary pattern	Ref.	0.85 (0.62–1.15)	0.56 (0.40–0.79)	0.001
Traditional Iranian dietary pattern	Ref.	1.26 (0.92–1.73)	0.91 (0.63–1.31)	0.678
high fat, high sugar dietary pattern	Ref.	1.18 (0.84–1.65)	1.41 (1.01–2.02)	0.047
Sensitivity analysis^b^				
Lacto vegetarian dietary pattern	Ref.	0.88 (0.61–1.26)	0.67 (0.45–0.99)	0.045
Traditional Iranian dietary pattern	Ref.	1.61 (1.11–2.34)	1.45 (0.96–2.18)	0.078
high fat, high sugar dietary pattern	Ref.	1.05 (0.71–1.53)	1.55 (1.04–2.32)	0.024
Sensitivity analysis^c^				
Lacto vegetarian dietary pattern	Ref.	0.96 (0.70–1.32)	0.69 (0.48–0.97)	0.042
Traditional Iranian dietary pattern	Ref.	1.21 (0.89–1.67)	0.78 (0.53–1.11)	0.204
high fat, high sugar dietary pattern	Ref.	1.14 (0.82–1.60)	1.43 (1.03–1.99)	0.033

^a^Sensitivity analysis was conducted on the total population after adjusting for age, sex, smoking, total energy intake, physical activity, body mass index, diabetes, and hypertension as well as triglycerides

^b^Sensitivity analysis was conducted after excluding participants with hypertension and diabetes (*n* = 1329) and adjusting for age, sex, smoking, total energy intake, physical activity, and body mass index

^c^Sensitivity analysis was conducted after excluding certain population who was at stake for unstable creatinine concentrations including pregnant and lactating, extremes physical activity, body mass index less than 18.5 and higher than 40 kg/m^b^ (*n* = 1493) and adjusting for age, sex, smoking, total energy intake, physical activity, body mass index, diabetes and hypertension

^d^To calculate the trend of OR across increasing tertiles of each extracted dietary pattern, we considered the tertiles categories as continuous variables (median values)

## Discussion

Following prospective analyses using data from a population-based study, we observed a trend of decreasing incident CKD risk with increasing intakes of a lacto-vegetarian dietary pattern among adults. In addition, participants who were in the highest tertile of the high fat, high sugar dietary pattern had a significant 49% increased odds of having incident CKD, independent of diabetes and hypertension.

In accordance with the our findings which lacto-vegetarian dietary pattern decreased risk of CKD, a study of diabetic patients from a cohort in Taiwan showed that extracted dietary pattern named fish and vegetable dietary pattern was independently associated with decreased adjusted means of creatinine levels [23]. A cross-sectional study from the Multi-Ethnic Study of Atherosclerosis (MESA) found that a dietary pattern including high intakes of whole grains, fruit, vegetables, and low-fat dairy foods was inversely associated with urinary albumin-to-creatinine ratio and the odds of microalbuminuria among adults without CVD, diabetes or microalbuminuria [11]. However unlike our findings, a study conducted on female nurses, aged 30–55 years, from the Nurses’ Health Study showed that higher adherence to the prudent dietary pattern (high intake of fruits, vegetables, legumes, fish, poultry, and whole grains) was not significantly associated with kidney function decline [10].

In previous studies we observed that both DASH diet and Mediterranean diet recommending foods and nutrients similar to the lacto-vegetarian dietary pattern characterized by high intakes of fruit, vegetables, low-fat dairy, and whole grains and low intakes of processed meat, have been shown to improve kidney function and decrease the risk of kidney damage [3, 4, 6, 24–26]. Several components of the DASH-style diet including fruits, whole grains, and nuts and legumes have also been associated with the decreased risk of CKD [4]. Higher compliance to the dietary pattern which is similar to the DASH diet could mediate decreased risk of CKD by modifying several cardio-metabolic risk factors, e.g. improved plasma lipid profiles, blood pressure, insulin sensitivity, oxidative stress, inflammation, and endothelial dysfunction [27–29]. Besides, the favorable effect of the lacto-vegetarian dietary pattern on kidney function has been illustrated by higher intakes of calcium, vitamin C, potassium, and magnesium, increases intakes of potassium and magnesium were associated with the lower dietary acid load. Recent studies suggest that dietary acid increment has an undesirable effect on kidney function [30]. Whereas, higher intakes of vegetables and fruits as main component of lacto-vegetarian dietary pattern had an inverse association with CKD [10, 11, 31]. Low-fat dairy, loaded in the lacto-vegetarian dietary pattern was inversely associated with ACR [11]. The amino acid and fatty acid composition of dairy foods in comparison with non-dairy might justify the different directions of associations. Furthermore, milk proteins, vitamin D, magnesium, and calcium may also contribute to these associations [32, 33].

Compared with participants in the lowest tertile of the high fat, high sugar dietary pattern, those in the highest one had 49% higher odds of incident CKD. Similar to our findings, the previous study among women from the Nurses Health Study showed the highest quartile of the Western dietary pattern (characterized by high intakes meat, processed meat, saturated fat, and sweets) compared with the lowest quartile has a 77% increased rapid eGFR decline after 11 years of follow-up [10]. Consistently, Shi et al. observed that a modern dietary pattern (high intake of fruit, soy milk, egg, milk and deep fried products) was associated with 50% decreased risk of CKD [12]. In this regard, studies found that higher consumption of red and processed meats, sugar-sweetened beverages, and sodium, which in combination were known as the Western dietary pattern, have been positively associated with CKD [11, 31, 34]. The Western dietary pattern is correlated positively with inflammatory markers such as CRP, interleukin 6, and E-selectin [2]; apparently these mediators may explain the direct association of the Western dietary pattern with kidney function impairment in those consuming more fat and meat [35].

From population-based studies that used factor analysis to identify dietary pattern, mostly two major dietary patterns were distinguished. The Prudent pattern is characterized by higher intakes of fruits, vegetables, legumes, whole grains, and sometimes poultry, and fish, whereas the Western pattern is characterized by higher intakes of saturated fats and processed and artificially sweetened foods, and salt. The former is consistently shown to have significant relation with lower risk of coronary heart disease, type 2 diabetes, and colorectal cancer, whereas the latter significantly elevated the risk of these diseases [36–38]. In the current study, the lacto-vegetarian dietary pattern was closely similar to the prudent pattern and the high fat, high sugar dietary pattern was consistent with western dietary pattern. In addition, it seemed that higher adherence to the lacto-vegetarian dietary pattern was accompanied by a healthier lifestyle including a decrease in smoking, BMI, and prevalence of diabetes and hypertension. In contrast, higher adherence to the high fat, high sugar dietary pattern was associated with increased smoking, BMI, and increased prevalence of hypertension. In this regard, previous studies extracting dietary pattern mostly described a healthy dietary pattern and high fat, high sugar dietary pattern. Nutrition transition of Iran as a subsequent issue of economic growth, urbanization, and industrialization push people toward the high fat, high sugar dietary pattern. Fast foods, sugar-sweetened beverage, and meat products high in saturated fat, sugar, and salt became more common in Iran.

Our findings revealed that there was no significant association between the traditional Iranian dietary pattern and incidence of CKD. Shi et al. observed that a traditional Southern dietary pattern (high intake of rice, pork, and vegetables, and low intake of wheat) was associated with 4.5-fold increased odds of prevalence of CKD. Netletton et al. illustrated that a dietary pattern which is similar to ours, high intakes of legumes, tomatoes, refined grains, high-fat dairy, and red meat was positively associated with albumin to creatinine ratio (ACR) [11]. The traditional dietary pattern of the Iranian population is identified mainly by high consumption of refined grain, egg, tea, potato, red meat, pickles, hydrogenated fat, and sugar as seen in previous studies, which used factor analysis to extract Iranian population dietary pattern [39–45]. In Iranian traditional dietary pattern, unhealthy food groups such as refined grains (white rice and bread), potatoes, and red meat were highly loaded; however, the presence of some healthy foods like legumes also loaded in this dietary pattern which could interact with other foods in the pattern to modulate consequent kidney dysfunction.

Although the association of major patterns identified through factor analyses with kidney function has been examined in several studies among American [10], European [11], and Chinese [12] populations, there is a lack of evidence in Iranian ones. Dietary patterns are likely to vary according to ethnic groups and cultures. It is thus necessary to replicate the results in diverse populations.

Limitations of this investigation need to be mentioned; first, as in most epidemiologic studies, our definition of CKD is based on a limited number of isolated creatinine measurements that were not repeated within three months to confirm a chronic reduction in GFR. Besides, although using creatinine estimated eGFR has a limitation because serum creatinine varied day-to-day (15.5–19.6%), most epidemiologic studies used serum creatinine for the definition of the CKD as it is cheap, technically simple and therefore easily applied for a large population measurements [5, 34]. Second, despite controlling for various confounders in our analysis, residual confounding due to unknown or unmeasured confounders such as socioeconomic factors cannot be excluded. However, the study’s noteworthy strengths, unlike previous studies, the current study provided data based on habitual dietary intakes in a population-based sample of participants, therefore, increasing the generalizability of its results.

## Conclusion

In conclusion, the high fat, high sugar dietary pattern was associated with a 49% increased and Lacto vegetarian dietary pattern diet was associated with a 37% decreased odds of for incidence of CKD after 6.1 years of follow-up, findings consistent with the hypothesis that particular aspects of diet may be important intervention targets to prevent incident CKD and subsequent kidney dysfunction. This present study also provides further information regarding reductions in kidney function and dietary patterns that may be more easily interpreted and followed by the general public.

## Additional file

Additional file 1: **Table S1.** Characteristics of participants who completed food frequency questionnaire (FFQ) in comparison to total population in the third phase of Tehran Lipid and Glucose Study (TLGS). (DOCX 13 kb)

## Abbreviations

CKD: chronic kidney disease
DASH: dietary approaches to stop hypertension
eGFR: estimated glomerular filtration rate
FFQ: food frequency questionnaire
