# Supplementary material for: Dietary pattern and incidence of chronic kidney disease among adults: a population-based study
Source: Nutr Metab (Lond). 2018 Dec 17;15:88. doi: 10.1186/s12986-018-0322-7 (PMC6296119; doi:10.1186/s12986-018-0322-7)
Supplement: Supplementary file 1 — Table S1. Characteristics of participants who completed food frequency questionnaire (FFQ) in comparison to total population in the third phase of Tehran Lipid and Glucose Study (TLGS). (DOCX 13 kb) [file 12986_2018_322_MOESM1_ESM.docx]

Table (supplementary) - Characteristics of participants who completed food frequency questionnaire (FFQ) in comparison to total population in third phase of Tehran Lipid and Glucose Study (TLGS)

|  | Total population | Participants with Completed FFQ |
| --- | --- | --- |
| Male (%) | 44.1 | 45.4% |
| Participant aged 19- to 70-year-old (%) | 82.3 | 76.7 |
| Body mass index (kg/m^2^) | 25.9±5.81 | 26.0±5.38 |
| Waist circumference (cm) | 86.7±14.9 | 86.7±15.8 |
| Academic education (%) | 20.1 | 25.3 |
| Smoker (%) | 11.6 | 12.8 |
